# Pupil dilation evoked by painful electrical stimulation is abolished during pain inhibition by distraction

**DOI:** 10.1016/j.jphyss.2025.100026

**Published:** 2025-05-16

**Authors:** Alice Wagenaar-Tison, Zoha Deldar, Antoine Bergeron, Benjamin Provencher, Stéphane Northon, Nabi Rustamov, Isabelle Blanchette, Sylvain Sirois, Mathieu Piché

**Affiliations:** a Department of Anatomy, Université du Québec à Trois-Rivières, Trois-Rivières, QC G9A 5H7, Canada; b Department of Psychology, Université du Québec à Trois-Rivières, Trois-Rivières, QC G9A 5H7, Canada; c School of Psychology, Université Laval, Québec, QC G9A 5H7, Canada

**Keywords:** Pain modulation, Attention, Nociceptive flexion reflex, Pupillometry, Electroencephalography

## Abstract

The aim of the present study was to examine the contribution of spinal and supraspinal processes to pain modulation by attention. It is hypothesized that pain inhibition by distraction is accompanied by reduced pain-evoked pupil dilation and cerebral activity, but no inhibition of the nociceptive flexion reflex (NFR), while pain anticipation is expected to increase pain perception and pain-related responses. Twenty healthy volunteers received 90 painful stimuli in control, distraction (mental arithmetic), and anticipation (visual cue) conditions. Anticipation did not modulate pain (p = .7), while distraction decreased pain significantly (p < .001). Moreover, pupil diameter increased 500–1000 ms post-stimulus in the control condition (p < .05), but this response was abolished by distraction. Distraction also decreased pain-related brain activity (high-gamma oscillations) (p = .004), but not the NFR (p = .3). These results suggest that pain inhibition by distraction is produced, in part, by supraspinal inhibition of nociceptive processes.

## Highlights

• Distraction reduces pain but not NFR, indicating supraspinal inhibition.
• Pain-evoked pupil dilation is abolished by distraction, lowering stimulus salience.
• High-gamma reduction reflects attentional redirection away from pain.
• Anticipation alters ERPs without changing pain perception.
• Combining NFR, EEG, and pupillometry separates spinal from supraspinal modulation.

## 1 Introduction

Limited attentional capacity theories posit that consciously processing all available information would overload the cognitive system [1], [2], [3], [4], [5]. Thus, the allocation of attention resources to process a stimulus would reduce the brain’s capacity to process other information [1], [2]. This has implications for pain perception. Indeed, the execution of a cognitive task that is not related to a painful stimulus significantly reduces pain perception and pain-related brain activity [6], [7], [8], [9], [10], [11].

The regulation of pain by cognition partly depends on cerebrospinal mechanisms [12], [13], [14], [15], [16], [17], [18]. In humans, cerebrospinal pain regulation may be indexed by changes in the amplitude of the spinal nociceptive flexion reflex (NFR). For example, decreased NFR amplitude during pain inhibition by distraction suggests that cerebrospinal inhibitory pathways are activated [14], [16]. However, the NFR may not be inhibited during pain inhibition by distraction, depending on the task [11], [19], [20], [21], [22]. For example, pain inhibition during an arithmetic task is not accompanied by NFR inhibition [11], [22]. This may reflect the inhibition of nociceptive inputs in ascending pathways after spinal nociceptive transmission occurred, by cortical or subcortical processes, but this remains to be clarified. As suggested previously, this may occur through the dorsolateral prefrontal cortex and its network (see [23] for review).

Painful stimuli are intrinsically salient and elicit an orienting response, which includes a brainstem-mediated pupil dilation [24]. This response may be used to examine the regulation of nociceptive inputs along the ascending nociceptive pathways above the spinal cord (supraspinal processes), particularly if the NFR is not modulated. Previous studies have shown an increase in pupil diameter following painful stimulation [25], [26], and a relationship between pupil diameter and experimental or clinical pain intensity [27], [28], [29], [30], [31], [32], [33]. According to these findings, pupil dilation evoked by pain is expected to decrease during pain inhibition by distraction. Without inhibition of the NFR, the reduced pain-evoked pupil dilation would suggest the involvement of supraspinal processes in pain inhibition. In contrast, attention to pain produced by an anticipatory signal is expected to preserve or increase pupil dilation evoked by pain, but this remains to be examined. The measurement of pupil diameter and the NFR during pain modulation may thus allow to tease apart spinal and supraspinal pain regulation processes. To complement the measure of spinal and brainstem processes, pain-related brain activity may be examined with electroencephalography. More specifically, the noxious electrical stimulation of the sural nerve that evokes the NFR also elicits event-related brain potentials (P45, N100 and P260) and spectral perturbations (2–10 Hz, 8–29 Hz, 30–60 Hz, and 61–90 Hz oscillations) [34], [35], [36], [37] that can be examined to assess pain-related brain activity.

The aim of the present study was to examine the contribution of spinal and supraspinal processes to pain modulation by attention. It is hypothesized that pain inhibition by distraction is accompanied by reduced pain-evoked pupil dilation and cerebral activity, but no NFR inhibition compared with a control condition. In contrast, anticipation of pain is expected to increase NFR amplitude, pain-evoked pupil dilation, cerebral activity, and pain perception, compared with a control condition.

## 2 Methods

### 2.1 Ethics approval

All experimental procedures conformed to the standards set by the latest revision of the Declaration of Helsinki and the guidelines of the International Association for the Study of Pain. All procedures were approved by the Research Ethics Committee of the Université du Québec à Trois-Rivières. All participants gave written informed consent, acknowledging their right to withdraw from the experiment without prejudice and received a compensation of $25 for their travel expenses, time, and commitment.

### 2.2 Participants

Twenty participants (14 women and 6 men; mean age ± SD: 24.1 ± 6.8 years-old) were recruited by advertisements on the campus of the Université du Québec à Trois-Rivières. Participants were included if they were between 18 and 45 years old with normal or corrected-to-normal vision. They were excluded if they reported acute or chronic pain, acute or chronic illness, psychiatric disorders, or took any medication or recreational drugs during the two weeks prior to the experiment. The twenty participants completed the study, and no data was excluded from the analysis.

### 2.3 Experimental design

This study relied on a within-subject design to examine the effects of distraction and anticipation on pain, pain-related anxiety, the nociceptive flexion reflex (NFR), pupil diameter and pain-related brain activity. After individual adjustment of stimulus intensity, participants received 90 painful electrical stimuli distributed equally in 3 different conditions: control, distraction, and anticipation (see *2.5 Experimental protocol*). Distraction was produced by a mental arithmetic task. Anticipation was produced by a visual cue presented 1 s before the painful stimulus, to increase attention to pain. The three experimental conditions were counterbalanced to avoid sequence order effects. The task lasted approximately one hour.

### 2.4 Painful electrical stimulation

Transcutaneous electrical stimulation (trains of 10 ×1 ms pulses at 333 Hz) was delivered with an isolated DS7A constant current stimulators (Digitimer Ltd., Welwyn Garden City, Hertfordshire, UK) triggered by a Grass S88 train generator (Grass Medical Instruments, Quincy, MA, USA). The stimulation was controlled by a script running in a stimulus presentation program (E-Prime2, Psychology Software Tools, Sharpsburg, PA, USA). The inter-stimulus interval varied randomly for all conditions, ranging from 6 to 8.2 s. The skin over the retromalleolar path of the right sural nerves was degreased and rubbed with alcohol. The skin was stimulated by a pair of surface electrodes (EL258, Biopac Systems, Inc., Goleta, CA, USA) with an inter-electrode distance of 2 cm. The NFR threshold was determined using the staircase method including three series of stimuli of increasing and decreasing intensity [38], [39]. Stimulus intensity was then adjusted individually to 120 % of the NFR threshold [38], [39], [40].

### 2.5 Experimental protocol

The experimental task was displayed on a computer screen using a stimulus presentation software (E-Prime2, Psychology Software Tools, Sharpsburg, PA). The task was divided into three conditions. In each condition, participants were instructed to keep their gaze on a central fixation cross. In the control condition, participants received 30 painful stimuli with no task. In the distraction condition, a mental arithmetic task was performed while the 30 painful electrical stimuli were delivered. The mental arithmetic task consisted of successive mental subtractions of 17 units beginning from 5000. A similar task was used in a previous study and was successful at producing the sensorimotor dissociation between pain and the NFR [41]. Participants were instructed to perform as many operations as possible with no errors. Every 5 painful stimuli, the fixation cross was replaced by a black screen and no painful stimulus was delivered to allow participants to report the result of their calculations. If the answer corresponded to a correct number (5000 minus a multiple of 17 units), participants could resume from that number. If the answer was incorrect, participants started over from 5000. After the answer was provided, the fixation cross was displayed, and the task resumed. In the anticipation condition, the fixation cross was replaced by a circle for 1 s, immediately before the painful electrical stimulus, cuing the upcoming stimulus. Each condition lasted between four and six minutes. Conditions were separated by a pause of at least 2 min and participants resumed when they were ready. The task lasted less than 30 min. The order of conditions was counterbalanced between participants to avoid sequence order effects.

### 2.6 Pain and pain-related anxiety ratings

After the last painful stimulus of a condition, participants were asked to rate the mean pain and pain-related anxiety verbally on two successive numerical rating scales (NRS) displayed horizontally on the computer screen. For pain intensity, the left and right extremities of the NRS were labelled as “0 - No pain and 100 - Worst pain imaginable” [42]. The left and right extremities of the NRS were labelled as “0 - No pain-related anxiety and 100 - Worst pain-related anxiety imaginable” [42].

### 2.7 Nociceptive flexion reflex

Electromyography (EMG) of the short head of the biceps femoris was recorded with a pair of surface electrodes, with a ground electrode on the tibial tuberosity (EL-508, Biopac Systems, Inc., Goleta, CA, USA). The EMG signal was amplified 2000 times, band pass filtered (10–500 Hz), sampled at 1000 Hz (Biopac Systems, Inc., Goleta, CA, USA) and stored on a personal computer for off-line analyses. The raw EMG recordings were transformed into the root-mean-square and the resulting signal was used to quantify the amplitude of NFR to each shock by extracting the integral value between 90 and 150 ms after stimulus onset. This amplitude was standardized within-subject for each shock using T scores, mean-centered at 50.

### 2.8 Electroencephalographic recordings

Electroencephalographic activity (EEG) was measured at Fz, Cz, Pz, C3 and C4 electrodes in accordance with the 10– 20 International system, with the ground at Fpz electrode and linked earlobes as reference. These electrodes were selected based on previous studies [4], [5], [6], [7], [8]. Indeed, somatosensory-evoked potentials (N100 and P260) and ERSPs (time-frequency analyses) are measured at Cz with sural nerve stimulation. Additional electrodes are useful to confirm that response amplitude peaks at Cz [4], [5], [6], [7], [8]. Impedance was kept below 10 kΩ for all electrodes. Eye movements and blinks were recorded using electrooculography (EOG) with electrodes placed lateral to the outer canthus of each eye, to the suborbital ridge, and below the right eye. The reference electrode was placed on the forehead. EEG and EOG signals were filtered using a hardware 0.1 - 500 Hz band-pass filter and sampled at 1000 Hz for offline analyses.

### 2.9 Event-related potentials

The continuous EEG was exported EEGLAB v2020.0 for offline analyses running in Matlab R2020b (The MathWorks, Inc., Natick, Massachusetts, USA). Raw data were re-referenced to the common average. Data were filtered with a digital finite impulse response band-pass filter (0.5–30 Hz) before segmentation into epochs, extending from −100 ms to + 2000 ms relative to the stimulus onset. Epochs were baseline corrected using the −100 to 0 ms window. An Infomax independent component analysis was applied using the in-built EEGLAB function Runica to identify and remove components associated with noise. ERP were identified based on their polarity and latency (P45; N100; P260) at Cz electrode. The mean amplitude of these components was calculated in time windows defined as follows: P45: 45–55 ms; N100: 90–120ms; P260: 280– 350 ms.

### 2.10 Time-frequency analyses

Event-related spectral perturbations (ERSP) were analysed for each condition. Data from Cz were filtered using a digital finite impulse response bandpass filter (1–100 Hz). Data were segmented into stimulus-locked epochs from −1600 to 2600 ms, with time 0 corresponding to the onset of the painful electrical stimulus. The signal was baseline corrected using the −700 to −200ms time window. A Morlet wavelet convolution was computed using the channel time–frequency options available in EEGLAB v.13.5.4b.7. Two hundred time points were generated, and 100 linearly spaced frequencies were computed from 1 to 100 Hz. Variable cycles were used for low and high frequencies, with 3 cycles for lowest frequencies and up to 15 cycles for highest frequencies. This variable number of cycles allows for the wavelet convolution method to provide a better frequency resolution at lower frequencies and a better temporal resolution at higher frequencies. For each participant, the time–frequency data of all trials were averaged for each condition separately, resulting in 3 average time–frequency maps. The mean power in 4 regions of interest were extracted: from 2 to 10 Hz between 150 and 400 ms; from 8 to 29 Hz between 300 and 1000 ms; from 30 to 60 Hz between 100 and 350 ms; from 61 to 100 Hz between 150 and 350 ms, based on previous EEG studies [34], [37], [43], [44], [45], [46], [47], [48].

### 2.11 Pupillometry data collection and analyses

Data were collected using a Tobii T120 eye tracker (Tobii Technology, Stockholm, Sweden), running at 60 Hz, with a built-in 34 × 28 cm screen (resolution set at 800 × 600 pixels, 60 Hz refresh rate), located in a dimly lit, soundproofed cubicle. Data was sampled at a frequency of 60 Hz. For lost samples due to eye blinks or missed tracking, pupil diameter was attributed an arbitrary value of −1. These missing samples were interpolated before the analyses[49], [50]. To limit variability, data were filtered using a low-pass digital filter with a sample frequency to cut the frequency ratio to 15. Phase drift was removed by applying the filter twice, forward then backward [51]. Using a cut-off frequency of 4 Hz (i.e., where pupil diameter variations changed direction 4 or more times per second) preserved the shape of the data but removed a low-frequency noise. This makes interpolation of missing samples more reliable [49] (also see [52]).

Because left and right pupil diameters were highly correlated when samples were available for both eyes, regression equations were computed for left on right pupil and right on left pupil. When a sample had missing data for only one eye, pupil data from the other eye were used for interpolation [49]. Otherwise, interpolation was linear from the average of the last three samples before the break to the average of the three samples after the break. If the missing sample sequence reached the end of the trial, the end of the linear interpolation process was the mean pupil dilation of that participant on that trial based on all samples without missing data. Pupil data from both eyes were than averaged at each sample, and the analyses were based on these mean pupil diameters [49].

For low-level implicit processes such as a nociceptive response, it was expected that peak pupil responses would be observed about 500 ms after noxious stimulus onset, before the relatively later higher-order cognitive processes related to the subjective experience of pain, which would add noise to the response relevant to this study [9], [10]. To accommodate anticipation pupil responses to noxious stimulation, and to restrict pupil analysis to implicit pain-related responses, we analyzed pupil diameter from 500 ms before the onset of noxious stimulation to 1000 ms after [9], [10].

### 2.12 Statistical analyses

All statistical analyses were conducted using Statistica v13.1 (Dell Inc., Tulsa, OK, USA), except for pupillometry data that were analyzed with Matlab R2020b (The MathWorks, Inc., Natick, Massachusetts, USA). All results are expressed as mean ± SD. Statistical threshold was set at p ≤ 0.05. Pain, pain-related anxiety, P45, N100 and P260 mean amplitudes and latencies, the ERP peak-to-peak amplitude and ERSP power in the 4 frequency bands were compared between conditions using Greenhouse-Geisser corrected repeated-measures ANOVAs. Since the design includes only three conditions, significant effects were decomposed with the Fisher post-hoc test to examine the effects of distraction and anticipation compared with control. Effect sizes are reported based on partial eta-squared (*η*^*2*^_*p*_). Pupillometry was analysed using functional data analysis (FDA, [53]), allowing t-tests in this instance to be expressed as a function of time rather than as a discrete value based on arbitrary time-bins [52].

## 3 Results

### 3.1 Pain and pain-related anxiety

**Fig. 1:**
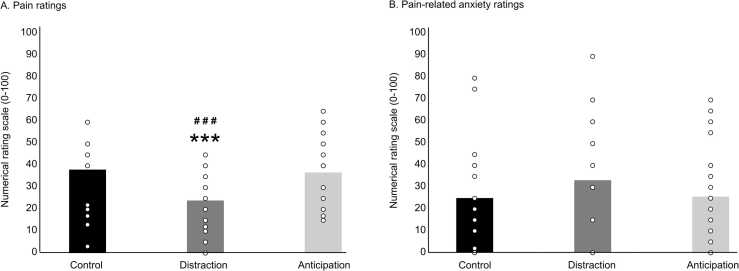
**Pain and pain-related anxiety.** (A) Pain ratings were significantly different between conditions (p < 0.001). The Fisher post hoc test revealed that distraction decreased pain compared with the control condition (p < 0.001) while anticipation produced no significant effect (p = 0.6). (B) Pain-related anxiety ratings were not significantly different between conditions (p = 0.3). Individual data are shown as open circles. * ** p < 0.001 compared with the control condition; # # # p < 0.001 compared with the anticipation condition.

Pain and pain-related anxiety ratings were compared between conditions with one-way repeated-measures ANOVA (see Table 1 and Fig. 1A-B). Pain ratings were significantly different between conditions (F_2,38_ = 16.2, p < .001, *η*^*2*^_*p*_ =.46; Control: 38.3 ± 18.0, Distraction: 24.1 ± 13.9, and Anticipation: 37.0 ± 16.1). The Fisher post hoc test revealed that distraction decreased pain compared with the control condition (p < .001, *η*^*2*^_*p*_ =.63), and the anticipation condition (p < .001, *η*^*2*^_*p*_ =.51), while anticipation produced no significant effect compared with the control condition (p = .6, *η*^*2*^_*p*_ =.01). As for pain-related anxiety, ratings were not significantly different between conditions (F_2,38_ = 1.3, p = .3, *η*^*2*^_*p*_ =.06; Control: 25.1 ± 23.9, Distraction: 33.3 ± 27.5, Anticipation: 25.8 ± 22.6).

**Table 1:** Descriptive data (mean ± SD).

	Control	Distraction	Anticipation
**Pain ratings (0 −100)**	38.3 ± 18.0	24.1 ± 13.9	37.0 ± 16.1
**Pain-related anxiety (0 −100)**	25.1 ± 23.9	33.3 ± 27.5	25.8 ± 22.6
**NFR (t-score)**	48.9 ± 7.1	52.3 ± 8.0	48.8 ± 9.4
**P45 (45 −55 ms)**			
Mean amplitude (µV)	0.7 ± 1.8	0.2 ± 1.9	0.5 ± 1.7
**N100 (90 −120ms)**			
Mean amplitude (µV)	−12.4 ± 6.2	−12.2 ± 6.1	−8.5 ± 4.2
**P260 (280 −350ms)** Mean amplitude (µV)	10.3 ± 6.6	9.2 ± 6.6	13.2 ± 6.9
**2 −10 Hz (dB)**	10.0 ± 3.7	10.5 ± 2.9	8.7 ± 3.5
**8 −29 Hz (dB)**	−2.2 ± 1.2	−1.8 ± 1.3	−2.7 ± 2.1
**30 −60 Hz (dB)**	1.9 ± 1.9	1.7 ± 1.2	1.1 ± 0.8
**61 −100 Hz (dB)**	2.7 ± 2.5	1.3 ± 1.5	2.0 ± 1.8

### 3.2 Nociceptive flexion reflex

**Fig. 2:**
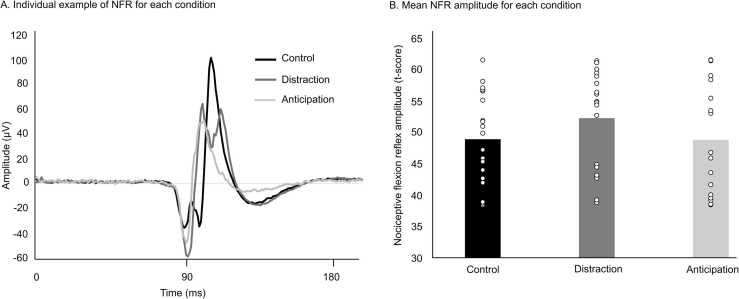
**Nociceptive flexion reflex.** (A) Individual example of the NFR in the three conditions. (B) NFR amplitude was not significantly different between conditions (p = 0.5). Individual data are shown as open circles.

An individual example of the NFR for each condition in the same participant is presented in Fig. 2A (raw data, before the transformation for quantification – see methods). The overlay shows a typical polyphasic response occurring at a latency of approximately 90 ms. NFR amplitude was compared between conditions with a one-way repeated-measures ANOVA (see Table 1 and Fig. 2B). In contrast to pain perception, NFR amplitude was not significantly modulated by distraction or anticipation (F_2,38_ =.8, p = .5, *η*^*2*^_*p*_ =.04).

### 3.3 Pupillometry

**Fig. 3:**
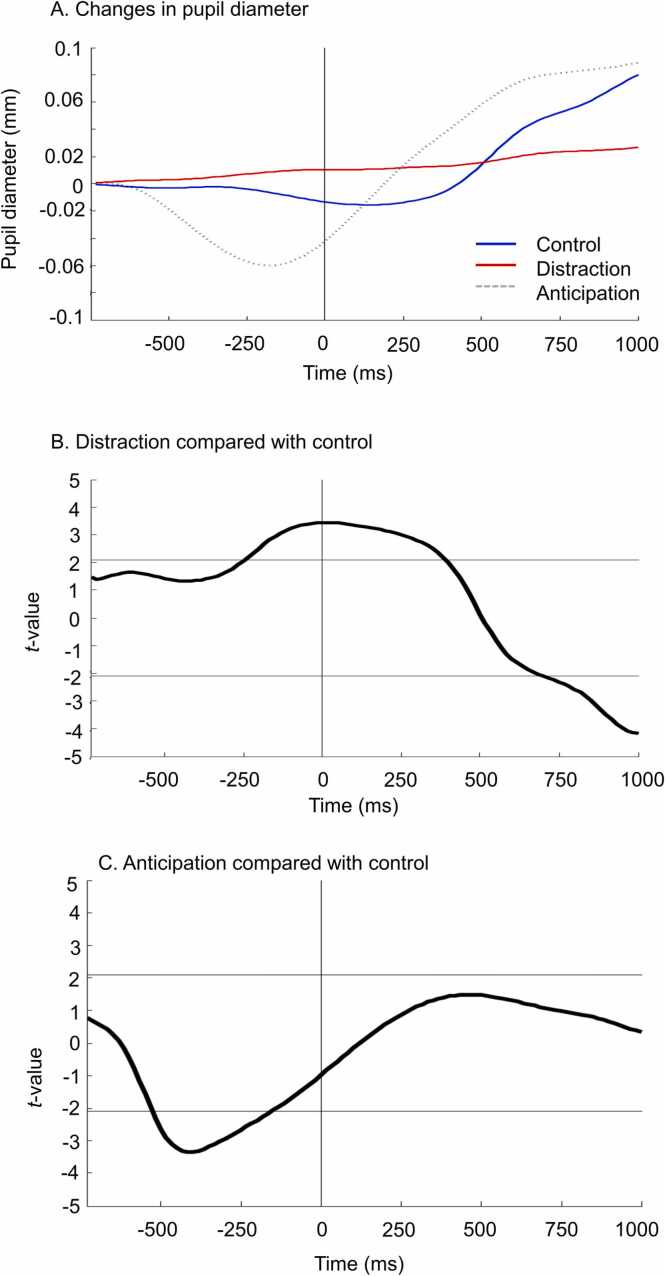
**Pupillometry.** (A) Changes in pupil diameter for the three conditions. The vertical gray line indicated the onset of the painful electrical stimulus. (B and C). Functional *t*-test between pairs of conditions. Horizontal lines represent the two-tailed critical *t*-value (p ≤ 0.05). Verticals gray lines represent the onset of painful electrical stimulus. Pain stimuli produced an increase in pupil diameter between 500 ms and 1000 ms post-stimulus in the control condition, but this response was abolished by distraction (see Fig. 3A-B). Moreover, anticipation produced by the visual cue decreased the pupil diameter and this effect was significantly different compared with the control condition between 500 ms and 200 ms pre-stimulus (Fig. 3A and C).

Changes in pupil diameter are shown in Fig. 3A and the functional *t*-tests between pairs of conditions are show in Fig. 3B-C. Pain stimuli produced an increase in pupil diameter between 500 ms and 1000 ms post-stimulus in the control condition, but this response was abolished by distraction (see Fig. 3A-B). Anticipation decreased pupil diameter compared with baseline, and this effect was significantly different compared with the control condition between 500 ms and 200 ms pre-stimulus (Fig. 3A and C).

Basal pupil diameter was also examined between conditions. On average, pupil diameter for the control, distraction, and anticipation conditions was 4.65 ± 0.65 mm, 4.86 ± 0.66, and 4.56 ± 0.65, respectively. No significant difference was observed between conditions (F_2,34_ = 2.5, p = 0.10, *η*^*2*^_*p*_ = 0.13).

### 3.4 Event-related potentials

**Fig. 4:**
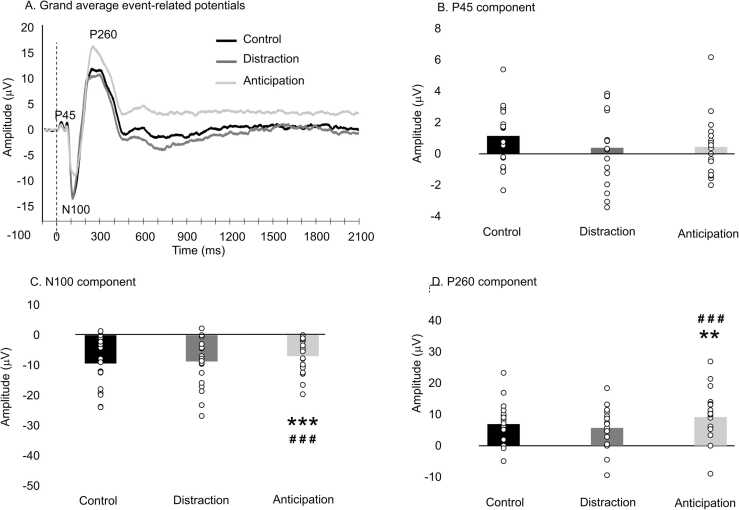
**Event-related potentials.** A. Grand average event-related potentials time-locked to electrical stimulation, recorded at Cz, for the three conditions. The vertical dashed line represents the onset of painful electrical stimulation. B. P45 mean amplitude (45–55 ms). The P45 amplitude was not significantly different between conditions (p = 0.4). C. N100 mean amplitude (90–120 ms). The N100 amplitude was significantly different between conditions (p < 0.001). The Fisher post hoc test revealed that anticipation decreased the N100 amplitude compared with the control condition (p < 0.002), but no significant difference was observed between distraction and the control condition (p = 0.8). D. P260 mean amplitude (280–350 ms). The P260 amplitude was significantly different between conditions (p < 0.001). The Fisher post hoc test revealed that anticipation increased the P260 amplitude compared with the control condition (p = 0.004), but no significant difference was observed between distraction and the control condition (p = 0.2). Individual data are shown as open circles. * * p < 0.01 and * ** p < 0.001 compared with the control condition; # # # p < 0.001 compared with the distraction condition.

The grand average of event-related potentials at Cz are presented in Fig. 4A. The mean amplitude and latency of the P45, N100 and P260 components were compared with repeated-measures ANOVAs (see Table 1 and Fig. 4B-D). The P45 mean amplitude was not significantly different between conditions (F_2,30_ = 0.9, p = 0.4, *η*^*2*^_*p*_ = 0.06). However, the N100 mean amplitude was significantly different between conditions (F_2,30_ = 11.2, p < 0.001, *η*^*2*^_*p*_ = 0.43). The Fisher post hoc test revealed that anticipation decreased the N100 amplitude compared with the control condition (p = 0.002, *η*^*2*^_*p*_ = 0.48) and the distraction condition (p < 0.001, *η*^*2*^_*p*_ = 0.53), but no significant difference was observed between distraction and the control condition (p = 0.8, *η*^*2*^_*p*_ = 0.003). Moreover, the P260 amplitude was significantly different between conditions (F_2,30_ = 11.5, p < 0.001, *η*^*2*^_*p*_ = 0.43). The Fisher post hoc test revealed that anticipation increased the P260 amplitude compared with the control condition (p = 0.004, *η*^*2*^_*p*_ = 0.44) and the distraction condition (p < 0.001, *η*^*2*^_*p*_ = 0.55), but no significant difference was observed between distraction and the control condition (p = 0.2, *η*^*2*^_*p*_ = 0.11). Regarding latencies, no significant difference was observed between conditions for any component (P45: F_2,30_ = 0.7, p = 0.5, *η*^*2*^_*p*_ = 0.05; N100: F_2,30_ = 2.1, p = 0.14, *η*^*2*^_*p*_ = 0.12; P260: F_2,30_ = 1.5, p = 0.3, *η*^*2*^_*p*_ = 0.09).

Although ERP waveforms were all baseline-corrected and baseline was around 0 for the three conditions, the waveform of the anticipation condition showed an upward shift from the N100 component, with no recovery to baseline. After careful examination of individual waveforms, all participants showed this upward shift. To further examine the effect of anticipation on pain-related brain activity, the N100-P260 peak-to-peak amplitude was compared between conditions with a repeated-measures ANOVA to exclude any bias due to a potential shift of the signal that may be unrelated to physiological changes. In contrast to the individual peaks, the peak-to-peak amplitude was not significantly different between conditions (F_2,30_ = 0.9, p = 0.4, *η*^*2*^_*p*_ = 0.06).

### 3.5 Event-related spectral perturbations

**Fig. 5:**
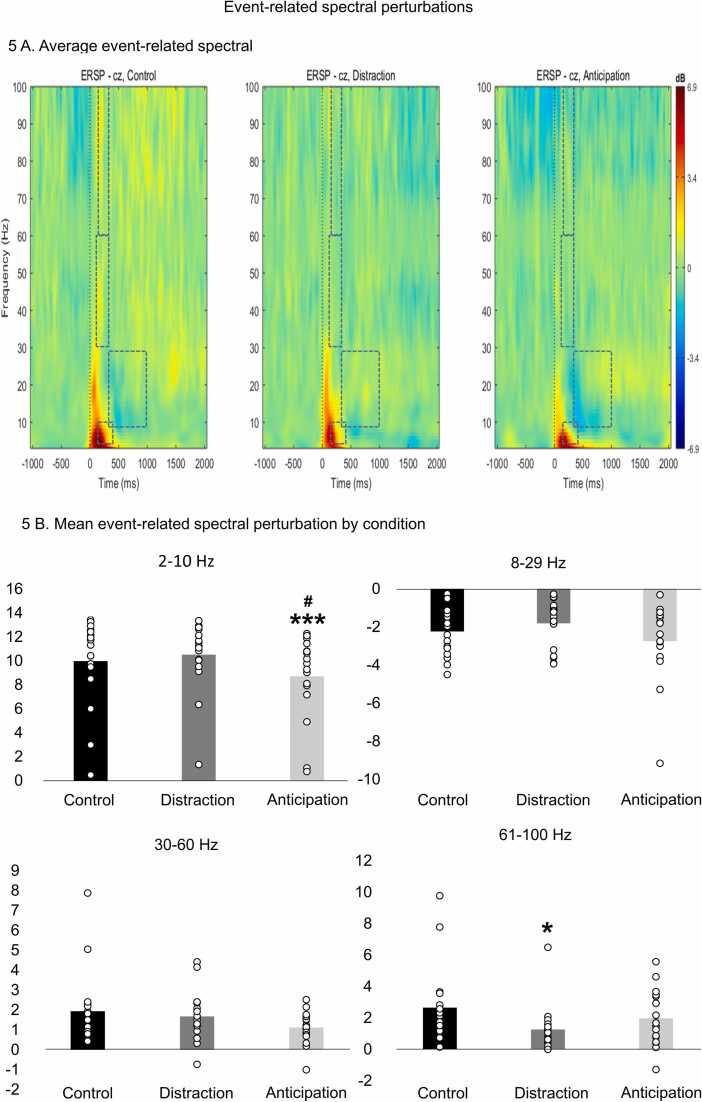
**Event-related spectral perturbations.** (A) Time-frequency maps at Cz for the three conditions. The regions of interest for each frequency band are represented by the dashed rectangles: 2–10 Hz (150–400ms), 8–29 Hz (300–1000ms), 30–60 Hz (100–350ms), and 61–100 Hz (150–350ms). (B) Comparison of mean power between conditions for each frequency band. For 2–10 Hz oscillations, power was significantly different between conditions (p = 0.025) and a Fisher post hoc test revealed that anticipation decreased 2–10 Hz oscillation power compared with the control condition (p < 0.001), but no significant difference was observed between distraction and the control condition (p = 0.4). For 8–29 Hz and 30–60 Hz oscillations, power was not significantly different between conditions (p = 0.12 and p = 0.14, respectively). For 61–90 Hz oscillations, power was significantly different between conditions (p = 0.014) and a Fisher post hoc test revealed that distraction decreased 61–90 Hz oscillation power compared with the control condition (p = 0.018), but no significant difference was observed between anticipation and the control condition (p = 0.10). * p < 0.05 and * ** p < 0.001 compared with the control condition; # p < 0.05 compared with the distraction condition.

ERSPs are shown in Fig. 5A. The regions of interest for each frequency band are represented by the dashed rectangles. For each frequency band, the oscillation power was compared between conditions with repeated-measures ANOVAs (see Fig. 5B). For 2–10 Hz oscillations, power was significantly different between conditions (F_2,32_ = 5.4, p = 0.025, *η*^*2*^_*p*_ = 0.25). The Fisher post hoc test revealed that anticipation decreased the 2–10 Hz oscillation power compared with the control condition (p < 0.001, *η*^*2*^_*p*_ = 0.56) and the distraction condition (p = 0.022, *η*^*2*^_*p*_ = 0.29), but no significant difference was observed between distraction and the control condition (p = 0.4, *η*^*2*^_*p*_ = 0.05). For 8–29 Hz and 30–60 Hz oscillations, power was not significantly different between conditions (F_2,32_ = 2.3, p = 0.12, *η*^*2*^_*p*_ = 0.13 and F_2,32_ = 2.3, p = 0.14, *η*^*2*^_*p*_ = 0.12, respectively). Lastly, for the 61–90 Hz oscillations, power was significantly different between conditions (F_2,32_ = 4.9, p = 0.014, *η*^*2*^_*p*_ = 0.23). The Fisher post hoc test revealed that distraction decreased the 61–90 Hz oscillation power compared with the control condition (p = 0.018, *η*^*2*^_*p*_ = 0.30) but not compared with the distraction condition (p = 0.10, *η*^*2*^_*p*_ = 0.16), and no significant difference was observed between anticipation and the control condition (p = 0.10, *η*^*2*^_*p*_ = 0.16).

## 4 Discussion

The aim of the present study was to examine the contribution of spinal and supraspinal processes to pain modulation by attention. The results revealed that distraction reduced pain, abolished the pain-evoked increase in pupil diameter, and decreased the power of pain-related high-gamma brain oscillations compared with the control condition. In contrast, the NFR was not significantly modulated by distraction. These results are consistent with previous findings [41] and suggest that pain reduction by distraction is produced, at least in part, by supraspinal inhibition of nociceptive inputs.

### 4.1 Pain inhibition by distraction

In the present study, distraction by the execution of an arithmetic task produced a strong inhibition of pain (37.1%, from 38.3 to 24.1 on the numerical pain rating scale), while the NFR was not significantly modulated. These results are consistent with previous studies indicating that distraction shifts attention away from pain, which inhibits pain perception [11], [12], [21], [54], [55], [56], [57], [58], [59], [60], [61], [62], [63], [64], [65]. The present results are also consistent with the neurocognitive model of attention to pain and the Limited attentional capacity theories [1], [2], [3], [4], [5], [66], [67], [68], [69]. These models propose that sensory signals, including pain-related information, compete for cognitive resources, where relevant signals necessary to goal-directed behavior are selected. Accordingly, when pain occurs, attention is drawn involuntarily to the noxious stimulus as a protective mechanism (attentional capture), but top-down mechanisms can reduce this effect and allow for prioritization of a goal-directed behavior, resulting in pain inhibition [41], [58], [63], [67]. According to the Limited attentional capacity theories, attentional resources could be saturated by task execution, and pain could be reduced by the limited processing of nociceptive inputs. In support of this interpretation, distraction decreased 61–90 Hz (high gamma) oscillation power compared with the control condition in the present study. Previous studies indicate that gamma oscillations are related to pain perception and stimulus saliency [37], [44], [45], [47], [70]. In addition, an interplay between pain-related and task-related visually-evoked gamma oscillations was shown previously, where an increase in pain-related gamma oscillations was associated with a decrease in task-related visually-evoked gamma oscillations [44]. Moreover, this cross-modal interaction was accompanied by attentional effects of pain, where the visual task performance was decreased. Therefore, the reduction in pain-evoked gamma oscillation power in the present study could reflect the allocation of attentional resources to the cognitive task, leading to reduced nociceptive processing [70], [71], [72]. This is consistent with the abolished pain-related increase in pupil diameter in the present study and the previously reported linear relationship between noxious electrical stimulation intensity and the mean change in pupil diameter [32]. This warrants future studies to examine the neural substrate underlying these effects, which may involve the DLPFC and it network [23]. Together, these results contribute to a better understanding of how the brain adapts to cognitive demands and distractors, which is crucial to enhance focus, attention, and the overall cognitive performance in different situations [73], [74].

In addition to the mechanisms discussed above, the context may influence the outcome of pain-cognition interactions. In experimental studies, the intensity of painful stimuli is adjusted individually, and participants know that stimuli are safe. This could favor goal-directed behaviors and reduce the impact of pain on cognition. In more ecological situations, a painful stimulus may be attributed a greater threat-value, capture attention, and prevent or alter task execution. Such effect may also be observed in experimental studies, where distraction may not always reduce pain perception, particularly when pain intensity is high, or in individuals are afflicted with chronic pain [75], [76], [77]. Moreover, cognitive engagement and effort are essential to reduce attentional capture by pain, and high task demands alone do not necessarily reduce pain perception [78]. With competing motivations (pain stimulus processing and goal-directed behavior), cognitive resources are allocated to the more favorable outcome [79]. Consequently, pain perception is inhibited only if other stimuli have more value [79]. In the present study, the strong inhibition of pain indicate that participants engaged in the arithmetic task, that the task was sufficiently demanding, and that it was attributed greater value compared with the painful stimuli. In contrast, pain may disrupt cognitive performance if cognitive engagement or task demands are not sufficient, or if pain is attributed greater value compared with the cognitive task. This may partly explain the bidirectional interaction between pain and cognition, as reported previously [9], [43], [44], [58], [62], [63], [80].

### 4.2 Regulation of the nociceptive flexion reflex

Pain is regulated by supraspinal and cerebrospinal processes [12], [13], [14], [16], [17], [18], [38], [58]. In humans, the NFR amplitude is used as an index of spinal nociceptive transmission [40], and may serve to investigate cerebrospinal pain regulation. In the present study, the inhibition of pain and pain-related responses by distraction was not accompanied by a reduction of NFR amplitude. This suggest that in the present experimental conditions, pain was inhibited by supraspinal processes and was not inhibited by cerebrospinal pathways. These results are consistent with those of a previous study in which pain inhibition during an arithmetic task was not accompanied by NFR inhibition, but rather facilitation [11]. Moreover, this interpretation is coherent with the abolished pain-related pupil dilation during distraction by the arithmetic task. Based on the association between pupil diameter and the activation of the salience brain network [81], this also suggests that the saliency of pain stimuli was decreased during distraction and is consistent with previous studies that showed a relationship between pupil diameter and pain intensity [26], [27], [28], [29], [30], [31], [32]. In line with the present results and previous findings, we propose that pain inhibition by distraction without NFR inhibition would allow for prioritization of goal-directed behaviors while preserving the protective function of a spinally mediated nociceptive reflex. However, it should be noted that the NFR comprises a sensory and a motor component that may be dissociated in certain conditions [38], and this may contribute to the present results and those from previous studies [11], [19], [20], [21], [22], [82]. This dissociation could be due to the facilitation or disinhibition of spinal motor neurons independently of spinal nociceptive neurons. In some contexts, this may be adaptive, where protective reflexes are maintained or enhanced, while nociceptive processing is inhibited to allow optimal cognitive performance. In the present study, it is not possible to determine whether the lack of NFR inhibition reflects a sensorimotor dissociation or whether both the sensory and motor components of the NFR were not inhibited. In humans, it remains challenging to tease apart these effects with the currently available experimental methods.

### 4.3 Regulation of pain and pain-related responses during pain anticipation

Anticipation of pain decreased the power of 2–10 Hz oscillations. This frequency band comprises theta and alpha oscillations. Theta oscillations (4–8 Hz) are involved in working memory, while alpha oscillations (8–13 Hz) are involved in attention and working memory [70], [72], [83]. The reduction in 2–10 Hz oscillation power by anticipation may reflect the allocation of cognitive resources to prepare for the upcoming pain stimulus. These findings align with the role of theta and alpha oscillations in sensory processing, attention, and cognitive processes [70], [72], [83], [84], [85]. Besides, anticipation reduced the N100 amplitude, an ERP component associated with early sensory information processing [86], and increased the P260 amplitude, which is related to cognitive processing of pain-related information [17], [87]. Together, these results indicate that both anticipation and distraction can modulate pain-related brain processes, but the effects are observed on different responses that affect behavior differently. One limitation for the interpretation of the N100 and P260 modulation by anticipation is the shift in EEG signal that was observed during this condition. The analysis of the N100-P260 peak-to-peak amplitude revealed no change in amplitude. Therefore, these results should be interpreted with caution and should be replicated. Also, the lack of pain modulation by anticipation could reflect that attention was already directed to the pain stimulus in the control condition and that the anticipatory signal was not sufficient to increase the allocation of further attention resources to the pain stimulus. This should be considered in the interpretation of the present findings and future study may produce different results if anticipation drives attention differently compared with the control condition.

## 5 Conclusion

In conclusion, the present results revealed that distraction reduced pain, abolished the pain-evoked increase in pupil diameter, and decreased the power of pain-related high-gamma brain oscillations compared with the control condition. In contrast, the NFR was not significantly modulated by distraction. This suggests that pain inhibition by distraction is produced, at least in part, by supraspinal inhibition of nociceptive inputs.

## Authors' contributions

Alice Wagenaar-Tison contributed to data analyses. Zoha Deldar wrote the first draft of the manuscript. Antoine Bergeron contributed to data collection and analyses. Benjamin Provencher and Stéphane Northon contributed to data analyses. Nabi Rustamov contributed to data collection. Isabelle Blanchette contributed to data interpretation and manuscript writing. Sylvain Sirois and Mathieu Piché contributed to study design, data analyses, data interpretation, and manuscript writing. Mathieu Piché wrote the final version of the manuscript and obtained funding for the study.

## CRediT authorship contribution statement

**Sylvain Sirois:** Writing – review & editing, Visualization, Validation, Supervision, Software, Resources, Methodology, Formal analysis, Data curation, Conceptualization. **Mathieu Piché:** Writing – review & editing, Visualization, Validation, Supervision, Software, Resources, Project administration, Methodology, Funding acquisition, Formal analysis, Data curation, Conceptualization. **Alice Wagenaar-Tison:** Writing – review & editing, Visualization, Formal analysis. **Zoha Deldar:** Writing – original draft. **Antoine Bergeron:** Investigation, Formal analysis. **Benjamin Provencher:** Formal analysis. **Stéphane Northon:** Formal analysis. **Nabi Rustamov:** Investigation. **Isabelle Blanchette:** Writing – review & editing.

## Consent for publication

The authors report no material that require permission and consent for publication. All authors read and approved the final version of this manuscript.

## Ethics approval and consent to participate

All experimental procedures conformed to the standards set by the latest revision of the Declaration of Helsinki and the guidelines of the International Association for the Study of Pain. All procedures were approved by the Research Ethics Committee of the Université du Québec à Trois-Rivières. All participants gave written informed consent, acknowledging their right to withdraw from the experiment without prejudice and received a compensation of $25 for their travel expenses, time, and commitment.

## Funding

This work was supported by a grant from the Natural Science and Engineering Research Council of Canada (MP).

## Declaration of Competing Interest

The authors declare the following financial interests/personal relationships which may be considered as potential competing interests: Mathieu Piche reports financial support was provided by Natural Sciences and Engineering Research Council of Canada. If there are other authors, they declare that they have no known competing financial interests or personal relationships that could have appeared to influence the work reported in this paper.

## Data Availability

The data that support the findings of this study are available from the corresponding author upon reasonable request.
